# Transcriptomic profiling of the telomerase transformed Mesenchymal stromal cells derived adipocytes in response to rosiglitazone

**DOI:** 10.1186/s12863-022-01027-z

**Published:** 2022-03-09

**Authors:** Moza Mohamed Al-Ali, Amir Ali Khan, Abeer Maher Fayyad, Sallam Hasan Abdallah, Muhammad Nasir Khan Khattak

**Affiliations:** 1 Department of Applied Biology, College of Sciences, University of Sharjah, Sharjah, 27272 UAE; 2 Human Genetics & Stem Cells Research Group, Research Institute of Sciences & Engineering, University of Sharjah, Sharjah, 27272 UAE; 3 Department of Molecular and Genetic Diagnostics, Megalabs Group, Amman, 11953 Jordan

**Keywords:** Telomerase-transformed mesenchymal stromal cells (iMSC3), Adipogenesis, Brown adipocytes, White adipocytes, Differentiation, mRNA-seq, Rosiglitazone, Transcriptomic analysis

## Abstract

**Background:**

Differentiation of Immortalized Human Bone Marrow Mesenchymal Stromal Cells - hTERT (iMSC3) into adipocytes is in vitro model of obesity. In our earlier study, rosiglitazone enhanced adipogenesis particularly the brown adipogenesis of iMSC3. In this study, the transcriptomic profiles of iMSC3 derived adipocytes with and without rosiglitazone were analyzed through mRNA sequencing.

**Results:**

A total of 1508 genes were differentially expressed between iMSC3 and the derived adipocytes without rosiglitazone treatment. GO and KEGG enrichment analyses revealed that rosiglitazone regulates PPAR and PI3K-Akt pathways. The constant rosiglitazone treatment enhanced the expression of *Fatty Acid Binding Protein 4 (FABP4)* which enriched GO terms such as fatty acid binding, lipid droplet, as well as white and brown fat cell differentiation. Moreover, the constant treatment upregulated several lipid droplets (LDs) associated proteins such as *PLIN1*. Rosiglitazone also activated the receptor complex *PTK2B* that has essential roles in beige adipocytes thermogenic program. Several uniquely expressed novel regulators of brown adipogenesis were also expressed in adipocytes derived with rosiglitazone: *PRDM16*, *ZBTB16*, *HOXA4*, and *KLF15* in addition to other uniquely expressed genes.

**Conclusions:**

Rosiglitazone regulated several differentially regulated genes and non-coding RNAs that warrant further investigation about their roles in adipogenesis particularly brown adipogenesis.

**Supplementary Information:**

The online version contains supplementary material available at 10.1186/s12863-022-01027-z.

## Background

Obesity is a growing health challenge worldwide. The global prevalence of obesity and overweight has increased to the pandemic levels [1]. According to the World Health Organization’s (WHO) recent data, more than 1.9 billion adults are overweight, and over 650 million are obese [2]. Obesity is a complex disorder characterized by an excessive or abnormal and pathological increase in fat deposition in adipose tissue. This excessive accumulation, in turn, increases the body mass index (BMI) above the normal range, causing deregulation of the metabolic balance and general health risks [2–4]. It is a major risk factor for many non-communicable and chronic diseases, including type 2 diabetes, dyslipidemia, hypertension, cardiovascular, musculoskeletal disorders, Alzheimer’s disease, and even some cancers. Moreover, it can amplify the risk they pose [1, 5].

Despite the availability of many different therapeutic approaches and interventions to control obesity, the problem remains unsolved. The conventional therapeutic approaches have many limitations which are pointing to the need for finding a new, novel, and innovative approach to treat obesity effectively [4, 6]. Stem cells of different types have shown their broad capacity and effectiveness in the treatment of different diseases through their differentiation potentials. Utilizing adipose-derived stromal cells through cell-based therapy seems a promising strategy to manage obesity and related syndromes [4]. However, further understanding of adipogenesis is required for the development of effective treatment [7].

Mesenchymal Stem Cells (MSCs) are multipotent cells that has the capacity of differentiating into a variety of mesodermal cells including adipocytes [7]. Therefore, MSCs play a vital role in obesity through the generation of adipocytes, and the differentiation is considered an in vitro model of obesity [7, 8]. Adipogenesis is characterized by sequential changes in the cell’s gene expression profile, primarily at the transcriptional level and then differential regulation of proteins [9]. Various early, intermediate, and late markers such as mRNAs and proteins are expressed as a result of activation by several groups of transcription factors, hormones, growth factors, and extracellular matrix (ECM) proteins [9, 10]. All of these modulators work in an ordered multistep process by transferring extracellular growth and differentiation signals and regulating the whole differentiation process intracellularly. MSCs will initiate to accommodate the spherical shape, enlarge and accumulate triglyceride droplets in their cytoplasm displacing the nucleus to the cell periphery, and acquire the biochemical characteristics of a mature adipocyte [11, 12]. The multistep process of adipogenesis is divided into two major phases [7]. The first phase is known as the determination or the commitment phase where the multipotent MSCs commit to the adipocyte lineage and appear as pre-adipocytes. The second phase is known as terminal differentiation. Here, the pre-adipocytes are converted to mature adipocytes acquiring the full characteristics and the necessary adipocyte-specific machinery [9, 13].

Adipose tissue is classically divided into two subtypes: Brown Adipose Tissue (BAT) and White Adipose Tissue (WAT) [9]. White adipocytes are the primary site of fat storage in the form of triacylglycerol in periods of energy excess, and the main fat metabolism orchestrator that works to release energy during energy deprivation [9, 10]. When the energy requirements exceed the energy reserves, the stored triacylglycerol is mobilized as free fatty acids and glycerol through lipolysis [14]. Brown adipocytes, on the other hand, serve to dissipate energy through thermogenesis rather than fat storage and are relatively scarce unlike the widely distributed white adipocytes [9, 10]. Given these facts, it is concluded that excess WAT is the main cause of obesity.

For effective prevention, management, and better therapeutic intervention of obesity, it is essential to study adipogenesis from progenitor cells to mature adipocytes and unravel the molecular mechanisms in such differentiation. This can be achieved by identifying the main signaling pathways and different genes that play a key role in the differentiation process. In adipose tissue, the *nuclear peroxisome proliferator-activated receptor γ (PPAR-γ)* is a ligand-activated transcription factor being the master regulator of BAT and WAT adipogenesis. It has vital roles in glucose and fatty acid metabolism [15]. Rosiglitazone is one of the thiazolidinediones drugs (TZDs) that was used as an anti-diabetic drug and is a *PPAR-γ* analog [15, 16]. As reported in our previous study, rosiglitazone enhanced adipogenesis by overexpression of the two transcription factors: *PPAR-γ* and *CCAAT/enhancer binding protein α (C/EBP-α)*. More specifically, brown adipogenesis was enhanced by the upregulation of *Early B Cell Factor 2 (EBF2)* and *Uncoupling protein 1 (UCP1)* [17]. We reported that rosiglitazone enhances brown adipogenesis in association with the upregulation of the MAP kinase and PI3 kinase pathways. However, a deeper understanding of genes regulation during adipogenic differentiation, particularly brown adipocytes, and the effects of rosiglitazone on the transcriptomes during the differentiation is needed to be unraveled.

Therefore, in this study, we investigated the transcriptomic profiles of iMSC3 and the differentiated adipocytes from iMSC3 in the presence and absence of rosiglitazone. This transcriptomic study confirmed our previous findings and further our understanding about the molecular processes that govern the adipogenic differentiation program of iMSC3, and the effects of rosiglitazone on the enhanced adipogenic differentiation, particularly brown adipogenesis.

## Results

### Rosiglitazone enhances the differentiation of iMSC3 cells into adipocytes

**Fig. 1:**
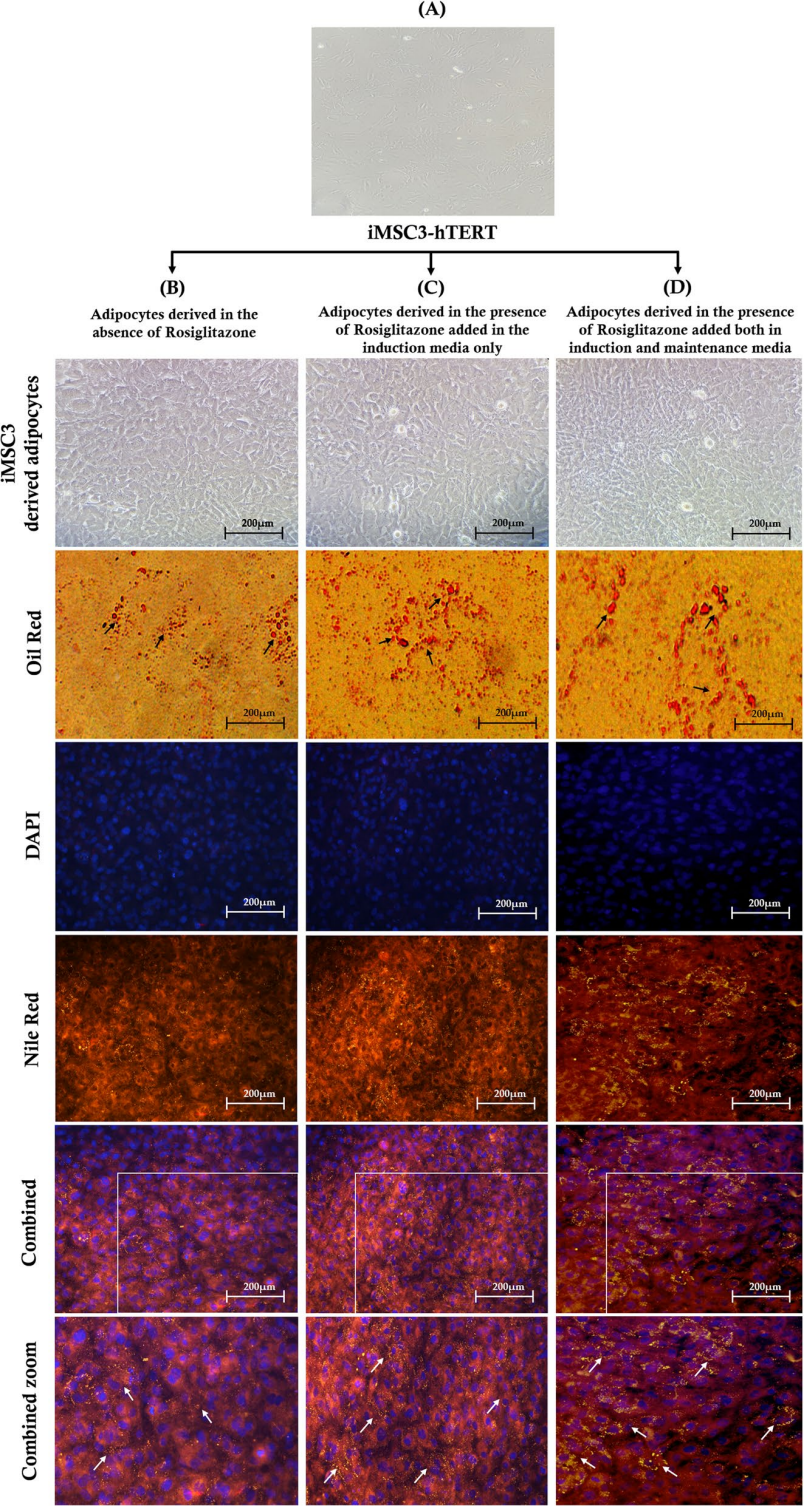
The effect of rosiglitazone on the differentiation of iMSC3 cells into adipocytes. **(A)** Fibroblast-like adherent mesenchymal stromal cells at 50–60% confluency, **(B)** mature differentiated adipocytes without rosiglitazone, **(C)** with rosiglitazone treatment in induction media only, and **(D)** both in induction and maintenance media. The iMSC3-derived adipocytes from control and treated groups were stained with nile red and Oil-O red to observe the lipid droplets accumulation and DAPI to visualize the nucleus. The observed morphological changes are characteristics of mature adipocytes. The lipid vesicles greatly increased and enhanced under rosiglitazone treatment

To unravel the role of rosiglitazone in adipogenesis, the iMSC3 were differentiated in vitro into adipocytes without and with the addition of 2 μM of rosiglitazone. The morphological changes at the beginning and the end of the differentiation cycles are demonstrated in (Fig. 1). The undifferentiated iMSC3 adherent cells have fibroblast like morphology (Fig. 1A). At the end of the differentiation cycle, the adipocytes from control (Fig. 1B) and treated cells (Fig. 1C,D) were stained with Oil-O red and nile red to specifically visualize the cytoplasmic LDs formation under different experimental conditions, and DAPI to stain the nucleus. The observed morphological changes in control and rosiglitazone treated cells are characteristics of mature adipocytes. The intensity of the stain increased in adipocytes with 2 μM rosiglitazone treatment (Fig. 1C,D) in comparison with the control adipocytes (Fig. 1B) as indicated by the arrows. The stain was most intense in adipocytes derived in the presence of rosiglitazone in both induction and maintenance media (Fig. 1D). The number of lipid vesicles greatly increased and enhanced with rosiglitazone treatment. This shows that rosiglitazone enhanced adipogenesis at the morphological level. Our previous study confirmed that rosiglitazone significantly increased the lipid content of the differentiated adipocytes through lipid quantification and increased the expression of *Fatty Acid Synthase (FASN)* gene responsible for triglycerides synthesis [17].

### mRNA sequencing, mapping and quantification

**Fig. 2:**
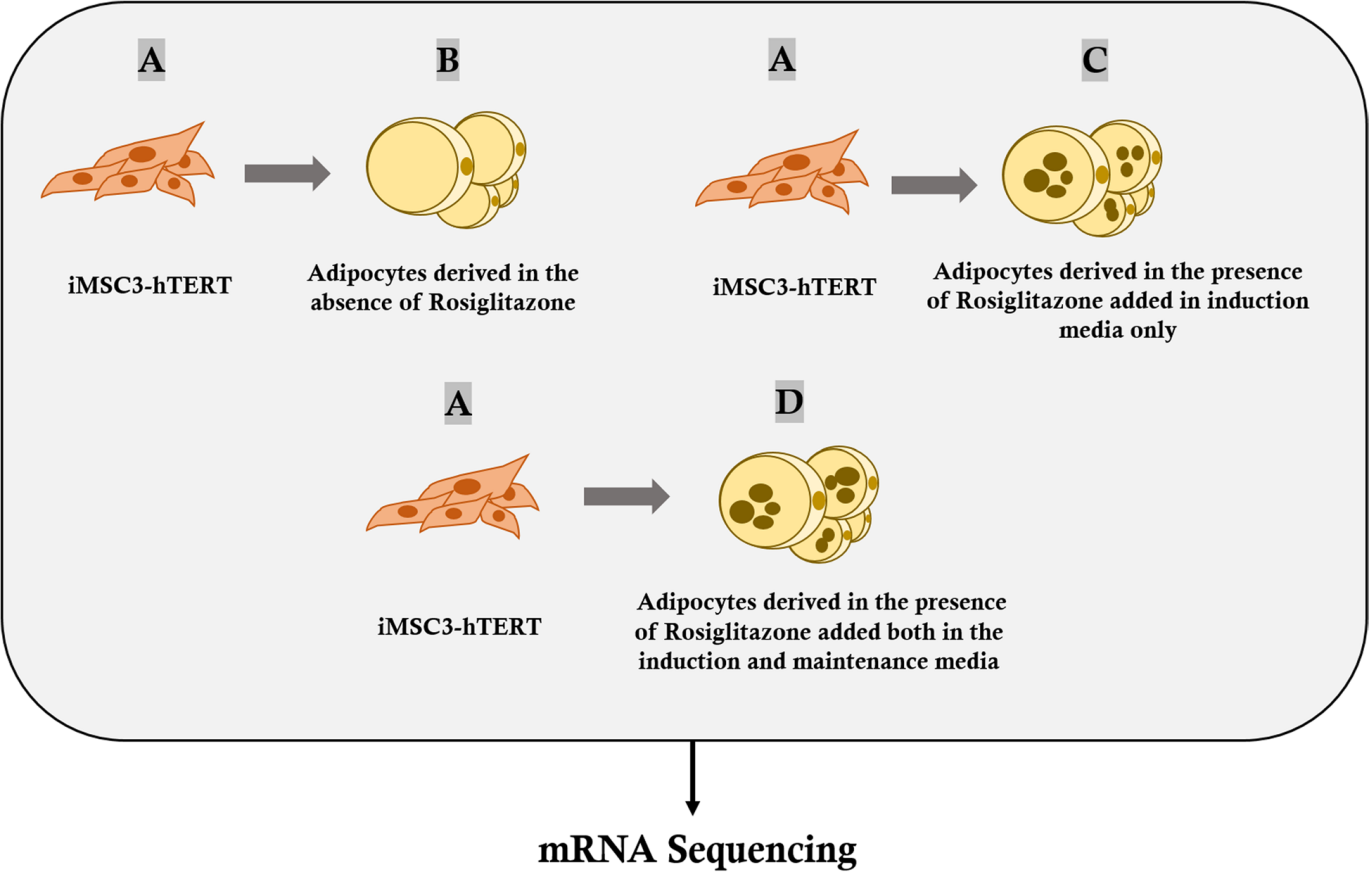
Graphical representation of the RNA-seq experimental plan. Undifferentiated iMSC3 cells **A**, iMSC3-derived adipocytes without rosiglitazone **B**, iMSC3-derived adipocytes under rosiglitazone treatment in induction media only **C**, iMSC3-derived adipocytes under rosiglitazone treatment both in the induction and maintenance media **D**. The total mRNAs from iMSC3 and the differentiated adipocytes were extracted for mRNA sequencing

To understand the molecular mechanism of rosiglitazone in enhancing adipogenesis at the transcriptomic level, RNA-seq was carried out. The sequenced mRNAs were obtained following the experimental plan depicted in (Fig. 2). To ensure the quality of downstream analysis, the sequencing raw reads were filtered to obtain clean reads by removing adaptor sequences or low-quality reads. The sequencing had effectively generated large numbers of high quality paired-end reads in all samples. All data quality is summarized in (Table S1). Spliced Transcripts Alignment to a Reference (STAR) software was used to map clean reads directly to the reference transcriptome for the differential expression gene (quantification) analysis. The summary of reads mapping to the reference genome is reported in (Table S2).

### Differential gene expression analysis

**Fig. 3:**
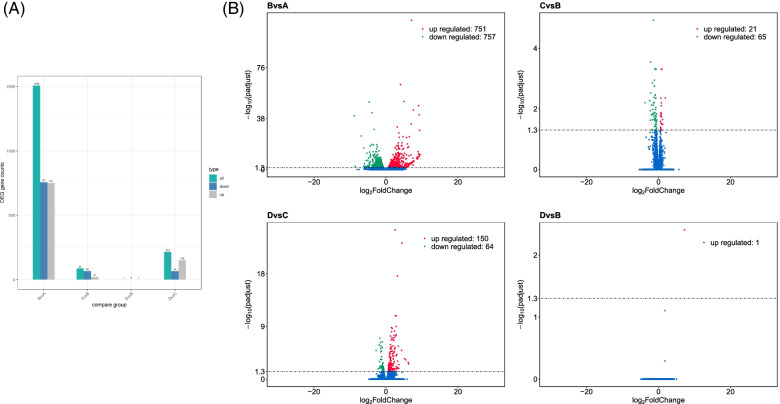
An overall presentation of differential gene expression analysis data. **A** Comparison of DEGs between undifferentiated iMSC3 A, adipocytes derived in the absence of rosiglitazone B, rosiglitazone added in induction media only C, and adipocytes derived in the presence of rosiglitazone added in both induction and maintenance media D. **B** Volcano plots demonstrating the overall distribution of DEGs. A high number of DEGs was found in B vs A compared to other groups studied. No significant differential expression was observed in D vs B except for one upregulated gene

The abundance of transcripts reflects gene expression level, which is calculated by the number of mapped reads and represented as Fragment Per Kilobase per Million mapped reads (FPKM) value. Read counts are proportional to gene expression level, gene length, and sequencing depth. The read counts obtained from gene expression analysis as FPKM values were used for the analysis of Differentially Expressed Genes (DEGs). The analysis was performed for each two comparison groups separately with biological replicates using the DESeq2 R package. A total of 1508 genes were found to be differentially expressed between undifferentiated iMSC3 and the fully differentiated adipocytes A vs B, among which 757 were downregulated and 751 were found to be upregulated. The genes are involved in the adipogenic differentiation of iMSC3 and consequently there is large transcriptomic changes between A and B. The comparison between the adipocytes derived in the presence of rosiglitazone added in induction media only with adipocytes derived without any addition of rosiglitazone C vs B revealed that 65 genes were downregulated and 21 upregulated giving a total of 86 DEGs. Furthermore, by comparing the transcriptomes of adipocytes derived with rosiglitazone in induction only and adipocytes derived in the presence of rosiglitazone in both induction and maintenance media C vs D, a total of 214 genes were found to be differentially expressed. Downregulated genes were 64, while the upregulated genes were 150. Surprisingly, only one significant differential expression was observed between fully rosiglitazone treated adipocytes and untreated adipocytes D vs B in *FABP4* gene (Fig. 3A). Volcano plots were used to infer the overall distribution of DEGs (Fig. 3B). The top 20 DEGs in each sequenced group are listed in (Table 1). The list of all differentially regulated genes is included in (Supplementary File 2).

**Table 1:** Summary statistics of the top 20 DEGs between all experimental groups studied

Comparison Group	Ensembl	Gene Symbol	Gene Type	Log2 Foldchange	Adjusted ***P*** value
**A vs B**	ENSG00000175899	A2M	protein_coding	9.48319179798626	8.17365800438721E-12
	ENSG00000162706	CADM3	protein_coding	9.363748	4.91E-30
	ENSG00000189058	APOD	protein_coding	9.295496	7.84E-11
	ENSG00000130600	H19	processed_transcript	9.225326	1.32E-41
	ENSG00000173432	SAA1	protein_coding	9.197134	5.16E-12
	ENSG00000109906	ZBTB16	protein_coding	9.16458	2.70E-10
	ENSG00000018625	ATP1A2	protein_coding	9.085414	1.92E-12
	ENSG00000105664	COMP	protein_coding	9.065493	1.85E-48
	ENSG00000157766	ACAN	protein_coding	8.901535	4.89E-14
	ENSG00000094963	FMO2	protein_coding	8.842061	9.96E-09
	ENSG00000242221	PSG2	protein_coding	−5.478177262	1.76E-07
	ENSG00000213030	CGB8	protein_coding	−5.611914079	0.018018551
	ENSG00000134321	RSAD2	protein_coding	−5.820959567	0.003985431
	ENSG00000280744	LINC01173	lincRNA	−5.910671503	0.021523417
	ENSG00000118785	SPP1	protein_coding	−5.971966402	3.65E-05
	ENSG00000231924	PSG1	protein_coding	−6.148461684	9.60E-17
	ENSG00000267399	AC006305.2	lincRNA	−6.148961014	0.002403624
	ENSG00000197632	SERPINB2	protein_coding	−6.850408513	8.79E-26
	ENSG00000187689	AMTN	protein_coding	−8.565848285	0.004588762
	ENSG00000196611	MMP1	protein_coding	−8.843058373	7.52E-41
**B vs C**	ENSG00000226145	KRT16P6	transcribed_unprocessed_pseudogene	1.906834	0.004413
	ENSG00000130487	KLHDC7B	protein_coding	1.177422	0.032238
	ENSG00000230479	AP000695.1	antisense	1.140401	0.022382
	ENSG00000249992	TMEM158	protein_coding	0.962648	0.000486
	ENSG00000011347	SYT7	protein_coding	0.932066	0.032238
	ENSG00000178860	MSC	protein_coding	0.832252	0.018459
	ENSG00000204941	PSG5	protein_coding	0.822275	0.049956
	ENSG00000171631	P2RY6	protein_coding	0.806492	0.006323
	ENSG00000251493	FOXD1	protein_coding	0.781888	0.000501
	ENSG00000197461	PDGFA	protein_coding	0.780789	0.014805
	ENSG00000166448	TMEM130	protein_coding	−1.720031175	0.023062466
	ENSG00000115457	IGFBP2	protein_coding	−1.921042358	0.049956389
	ENSG00000027644	INSRR	protein_coding	−1.934781349	0.007549294
	ENSG00000112936	C7	protein_coding	−1.984568888	0.015663029
	ENSG00000116690	PRG4	protein_coding	−2.147330739	0.002988794
	ENSG00000109906	ZBTB16	protein_coding	−2.19922063	0.000284527
	ENSG00000230712	GGTLC4P	unprocessed_pseudogene	−2.265300867	0.011550051
	ENSG00000035664	DAPK2	protein_coding	−2.269291912	0.023766748
	ENSG00000145358	DDIT4L	protein_coding	−2.46254331	0.005384331
	ENSG00000064300	NGFR	protein_coding	−3.74063749	0.006322863
**B vs D**	ENSG00000170323	FABP4	protein_coding	7.220661624	0.003922
**C vs D**	ENSG00000184811	TRARG1	protein_coding	6.30441031	0.002390337
	ENSG00000170323	FABP4	protein_coding	6.181716427	0.001357736
	ENSG00000187288	CIDEC	protein_coding	5.526394468	0.000503077
	ENSG00000142973	CYP4B1	protein_coding	5.16623179	0.000315358
	ENSG00000186191	BPIFB4	protein_coding	4.445982062	6.01E-24
	ENSG00000064300	NGFR	protein_coding	4.322344932	1.25E-05
	ENSG00000069122	ADGRF5	protein_coding	3.605555324	0.003274355
	ENSG00000241644	INMT	protein_coding	3.565028765	0.015203987
	ENSG00000115468	EFHD1	protein_coding	3.429678334	2.21E-06
	ENSG00000166819	PLIN1	protein_coding	3.275438842	0.000873093
	ENSG00000013297	CLDN11	protein_coding	−1.468389004	5.93E-06
	ENSG00000170961	HAS2	protein_coding	−1.527061186	0.000424167
	ENSG00000165118	C9orf64	protein_coding	−1.529325645	5.64E-07
	ENSG00000250038	AC109588.1	lincRNA	−1.665267793	0.006007246
	ENSG00000138316	ADAMTS14	protein_coding	−1.696240088	9.84E-08
	ENSG00000255364	SMILR	lincRNA	−1.803735522	0.000992088
	ENSG00000235513	AL035681.1	antisense	−1.807650771	0.012265022
	ENSG00000172061	LRRC15	protein_coding	−1.885245728	1.49E-06
	ENSG00000139629	GALNT6	protein_coding	−2.142177667	0.016769417
	ENSG00000165495	PKNOX2	protein_coding	−2.721732704	1.24E-05

### Co-expression analysis

**Fig. 4:**
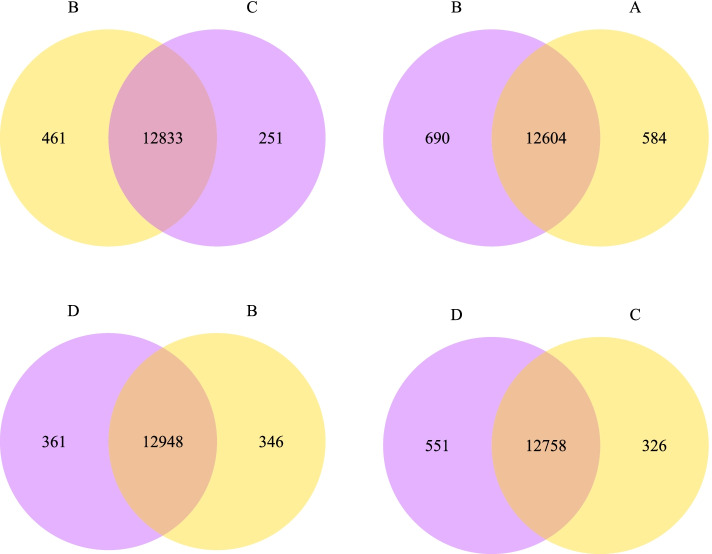
The Coexpression Venn diagram presenting the number of uniquely expressed genes in each group and the number of coexpressed genes in comparison with amongst the groups: undifferentiated iMSC3 **A**, adipocytes derived in the absence of rosiglitazone **B**, rosiglitazone added in induction media only **C**, and adipocytes derived in the presence of rosiglitazone added in both induction and maintenance media **D**

The co-expression Venn diagram presents the number of genes that are both uniquely and commonly expressed within each group comparison. Comparing undifferentiated iMSC3 A and the derived adipocytes in the absence of rosiglitazone B, a total of 584 genes were found to be uniquely expressed in A and 690 genes in B sharing 12,604 genes, including many involved in the adipogenesis. When the control group B is compared to adipocytes derived in the presence of rosiglitazone added in induction media only C, the number of co-expressed genes obtained is 12,833. Notably, group B has more unique genes than C, having a total of 461 and 251 genes, respectively. On the other hand, when C transcriptome is compared to adipocytes derived in the presence of rosiglitazone added in both induction and maintenance media D, the analysis demonstrates that group D has 551 unique genes compared to 326 genes for C. Finally, the comparison of group B with D yields a total of 12,948 co-expressed genes. Group D has 361 uniquely expressed genes while B showed only 346 genes. Overall, the later pair compared showed a higher number of uniquely expressed genes (Fig. 4), in contrast to the number of DEGs within the group. The list of all uniquely expressed genes is included in (Supplementary File 3).

### GO and KEGG enrichment analysis of DEGs enriched significant signaling pathways under rosiglitazone treatment

**Fig. 5:**
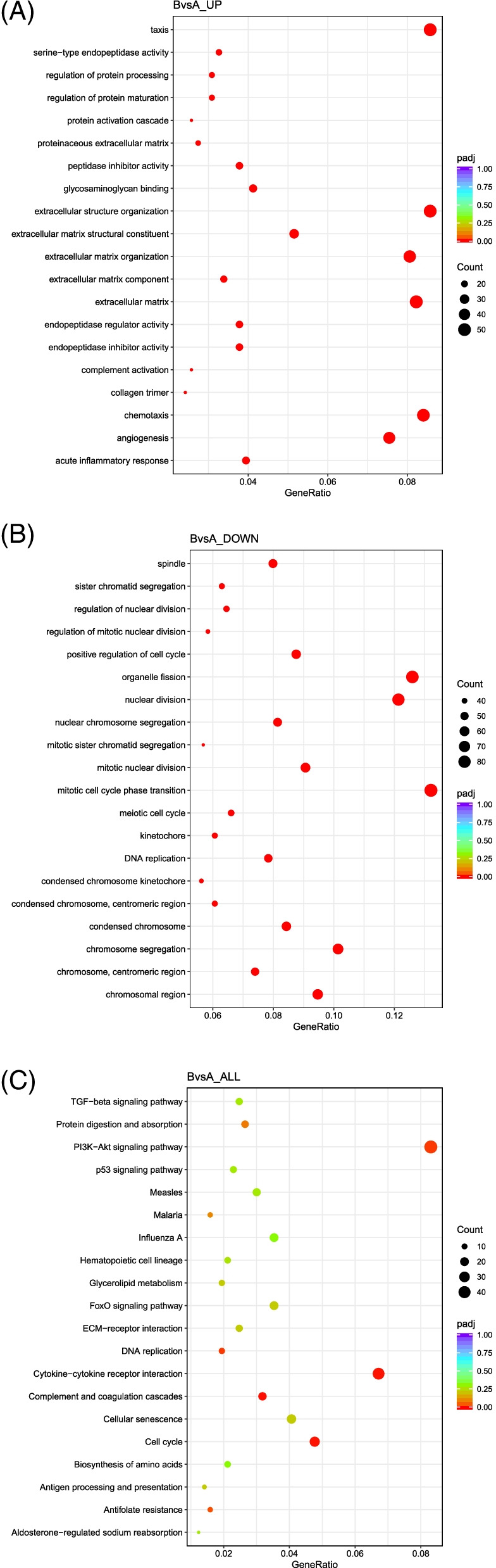
Top 20 enriched GO terms and KEGG pathways in the DEGs between iMSC3 and the derived adipocytes. **A** 20 most significantly upregulated GO terms. **B** 20 most downregulated GO terms. GO terms are shown in the y axis and the corresponding gene ratio on the x axis. **C** KEGG enrichment analysis. Significantly enriched pathways are presented in the y axis and the corresponding gene ratio on the x axis. The color scale represents the adjusted *P* value for each enriched term and pathway

To functionally classify the differentially regulated genes and to identify their involvement in metabolic pathways, GO & KEGG enrichment analyses were performed. Through enrichment analysis of the DEGs, significant biological GO terms or pathways were found to be enriched amongst the different groups. GO and KEGG enrichment analyses were performed using ClusterProfiler software with *P* value < 0.05. By comparing the DEGs in undifferentiated iMSC3 with fully differentiated adipocytes A vs B, 574 significant GO terms and 6 KEGG pathways were obtained [18]. The main 20 GO terms that are enriched during the iMSC3 differentiation into adipocytes B are given in (Fig. 5A&B). According to the results, DEGs between these two groups were mainly enriched in chromosomal assembly, cell cycle pathways, and ECM related genes. The main downregulated GO terms were associated with mitosis, including mitotic sister chromatid segregation and positive regulation of cell cycle. Pointedly, KEGG enrichment analysis revealed the presence of many significant signaling pathways including PI3K-Akt (Fig. 5C).

**Fig. 6:**
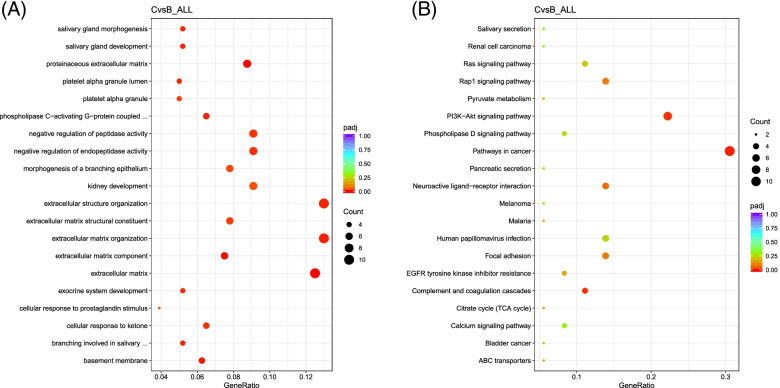
Dot plot of the most significant GO terms and KEGG pathways enriched in the DEGs between adipocytes derived in the absence of rosiglitazone B and rosiglitazone added in induction media only C. **A** Significantly enriched GO terms are shown in the y axis and the corresponding gene ratio on the x axis. **B** KEGG enriched pathways are presented in the y axis and the corresponding gene ratio on the x axis. Only three KEGG pathways were significantly enriched, among which PI3K-Akt pathway was found

The main GO and related terms enriched in the group C vs B were ECM related terms, cellular response to prostaglandin stimulus, and phospholipase G-protein activating protein (Fig. 6A). KEGG enrichment analysis identified regulation of PI3K-Akt pathway (Fig. 6B). This pathway is regulated with rosiglitazone treatment as reported in our previous study [17].

**Fig. 7:**
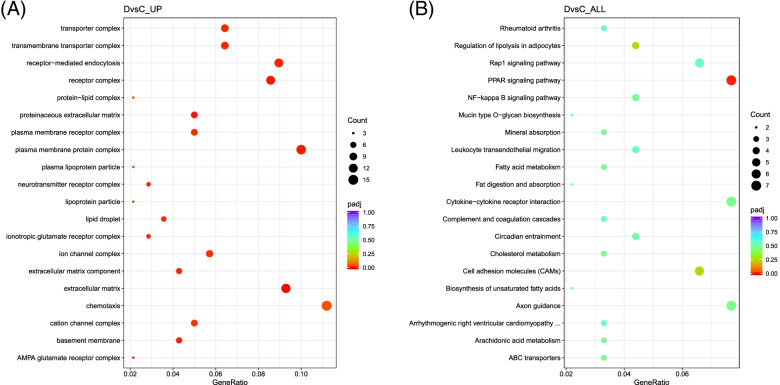
GO terms and KEGG pathways enriched in the DEGs between adipocytes derived with rosiglitazone added in induction media only C vs adipocytes derived with rosiglitazone present in both induction and maintenance D. **A** Significantly enriched GO terms that appeared to be upregulated in D. GO terms are shown in the y axis and the corresponding gene ratio on the x axis. **B** KEGG enrichment analysis showing the only significant enrichment of PPAR signaling pathway

The main and related GO terms in C vs D enriched in the upregulated genes were lipid droplet, plasma membrane receptor complex, lipoprotein particle, protein−lipid complex, and many ECM related genes (Fig. 7A). These enriched terms do indicate that the rosiglitazone induces brown adipogenesis. KEGG pathway enrichment indicates that PPAR signaling pathway is upregulated in D due to rosiglitazone treatment (Fig. 7B).

**Fig. 8:**
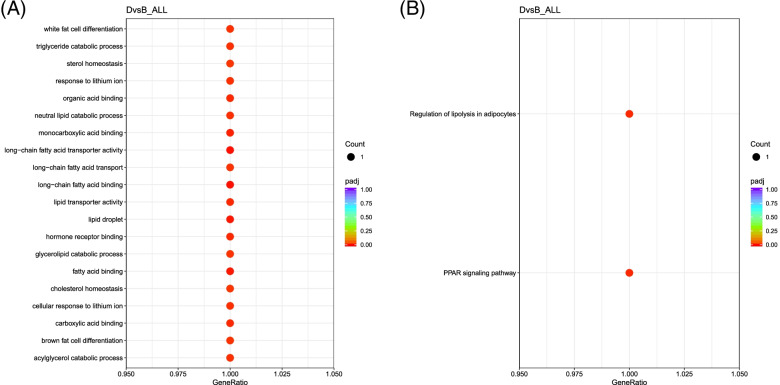
GO terms and KEGG pathways enriched in the DEGs between untreated adipocytes B vs adipocytes derived in the presence of rosiglitazone in the induction and maintenance media D. **A** The main significantly enriched GO terms. **B** Dot plot of KEGG enrichment analysis showing the significant enrichment of PPAR signaling pathway and the regulation of lipolysis in adipocytes due to *FABP4* expression in D

The GO terms enriched in adipocytes derived with the addition of rosiglitazone in both induction and maintenance media D in comparison to adipocytes B were due to the expression of the *FABP4*. The main GO terms enriched include fatty acid binding, lipid droplet, as well as white and brown fat cell differentiation (Fig. 8). This indicates that *FABP4* is upregulated by the constant presence of rosiglitazone and is the reason for enhancing the differentiation of brown adipogenesis at the transcription level in hand with other uniquely expressed genes or non-coding RNAs in D. The details of GO and KEGG enrichment analyses are included in (Supplementary File 4 and File 5), respectively.

### RNA-Seq validation by qRT-PCR

**Fig. 9:**
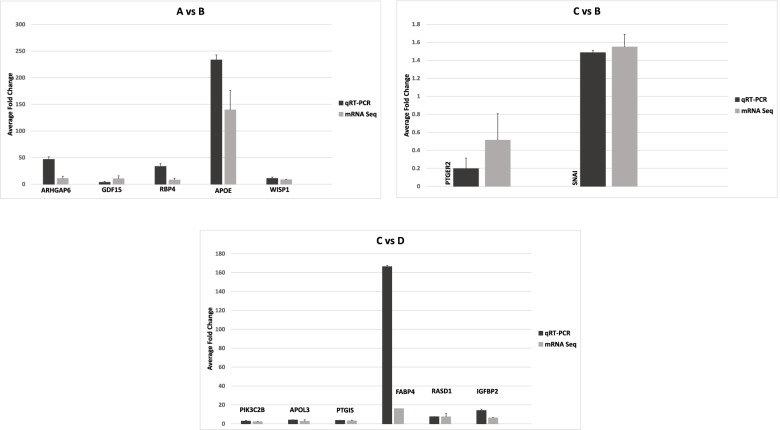
Comparison of average fold changes of selected genes in each experimental group obtained by qRT-PCR and mRNA sequencing: undifferentiated iMSC3 **A**, adipocytes derived in the absence of rosiglitazone **B**, rosiglitazone added in induction media only **C**, and adipocytes derived in the presence of rosiglitazone added in both induction and maintenance media **D**. Both RNA-seq and RT-qPCR showed same differential expression pattern. All fold changes obtained by qRT-PCR are statistically significant with *P* < 0.05

To confirm the differential gene expression data obtained by RNA sequencing, the expression of 13 genes were analyzed by qPCR. The genes were selected after performing DEGs analysis based on their relevance to adipogenesis. Overall, both RNA-seq and RT-qPCR showed same pattern of differential expression. The differential expression fold changes estimated by RT-qPCR for all 13 genes tested and the log transformed RNA-seq expression values (log2 fold change) were corresponding (Fig. 9).

## Discussion

In this study, we investigated the changes in the transcriptome profiles of terminally differentiated adipocytes derived, with and without rosiglitazone, from the undifferentiated iMSC3 by mRNA-Sequencing. Vast transcriptomic changes were associated with the differentiation of iMSC3 into adipocytes. The rosiglitazone treatment also regulated several genes that enhanced the adipogenic differentiation, specifically brown adipocytes.

The comparison of undifferentiated iMSC3 A to adipocytes B revealed the upregulation of many adipocytes markers confirming the successful adipogenic differentiation. The list included the novel adipocytokine *Retinol Binding Protein 4 (RBP4)* that is known to be associated with obesity, insulin resistance, and cardiovascular diseases [19]. Several studies reported increased *RBP4* expression with increased adipose tissue mass and its elevated serum level in obese human subjects [20]. *Growth Differentiation Factor 15 (GDF15)* is another adipokine that regulates lipid and glucose metabolism, increases insulin sensitivity, and appears to be important in maintaining body weight and preventing chronic inflammation [21, 22]. Several studies showed that *GDF15* levels are increased in patients with obesity and diabetes [21, 23].

GO terms enrichment analysis of adipocytes B in comparison to iMSC3 revealed the upregulation of ECM related genes (Fig. 5A). The ECM is a complex network composed of different proteins, proteoglycans, and polysaccharides [24, 25]. The changes in the ECM during adipogenesis are fundamental for the morphological transformation of the cells from a fibroblastic like structure to a spherical shape [24, 26]. Moreover, ECM remodeling and reorganization is essential for the regulation of adipogenesis by altering the expression of adipogenic genes, as well as for the enlargement of the existing adipocytes and the formation of new ones [26]. The ECM also provides strong external support for the mature adipocytes under strong mechanical stress; due to stored fats as triglycerides [25]. Consequently, the more mature adipocytes store fats, the more the ECM is expanded to accommodate this increase in cell volume [27]. Hence, a significant upregulation of ECM related GO terms in adipocytes B compared to undifferentiated iMSC3 cells A was observed. ECM synthesis, composition, and remodeling are structured based on the requirements for differentiation and maintaining the balance between flexibility and integrity of the tissue [28]. The downregulated GO terms in adipocytes B were chromosomal assembly and cell cycle related genes (Fig. 5B). It has been previously described that MSCs undergo a mitotic clonal expansion during early adipogenesis followed by growth arrest and adipogenic commitment [29, 30]. Proliferation related genes such as *cyclin D1 (CCND1)*, the cell cycle master regulator *cyclin dependent kinase 1 (Cdk1)*, *cell division cycle 6 (CDC6)*, *cyclin A2 (CCNA2)*, *polo like kinase 2 (PLK2)* were clearly downregulated in differentiated adipocytes, confirming that reduced proliferative activity promotes adipogenesis as described in a previous study [30]. KEGG analysis unveiled few significant signaling pathways (Fig. 5C). PI3K-Akt signaling pathway is known to play a fundamental role in cellular processes including lipid metabolism and glucose homeostasis, protein synthesis, and cell proliferation and survival. This pathway is thought to promote lipid biosynthesis and inhibits lipolysis [31]. Evidently, this pathway was upregulated in adipocytes B. Furthermore, the transcriptome analysis of uniquely expressed genes in adipocytes B revealed many other TFs including some that are linked to adipogenesis: *TFAP2E* [32], *POU5F1 (Oct4)* [33–35], *ZBTB16* [36, 37], *EGR2* [38], and *MAFB* [39]. These TFs along with many other genes and non-coding RNAs uniquely expressed in adipocytes warrant further investigation to find their rules in adipogenesis or obesity (Supplementary File 3). Overall, the upregulation of these markers confirms iMSC3 differentiation into adipocytes. These vast transcriptome changes in the derived adipocytes B indicate that the differentiation is associated with enormous transcriptomics changes.

To further confirm the effect of rosiglitazone treatment on the enhancement of adipogenesis, the transcriptome profiles among B, C and D were analyzed. Among the significantly enriched gene between C vs B is *phosphoenolpyruvate carboxykinase 2 (PCK2)* which was upregulated in C. *PCK2* is a transcriptional inducer that directs the activation of *phosphoenolpyruvate carboxykinase (PEPCK-C)* enzyme during adipogenesis [40, 41]. This enzyme catalyzes the glyceroneogenesis pathway in adipocytes that is important for fatty acid re-esterification and reduced fatty acid release and is robustly expressed in brown adipose [41, 42]. As *PCK2* is a peroxisome proliferator activated receptor γ response element (PPRE), it is also activated by thiazolidinediones [40]. This might explain the upregulation of *PCK2* in adipocytes treated with rosiglitazone C. Within the uniquely expressed genes in adipocytes C vs B, the transcription factor *PBX/knotted 1 homeobox 2 (PKNOX2)* was present. This TF was reported as a novel potential regulator of browning in the bulk RNA-seq of five different mouse strains [43]. The same gene was upregulated in C compared to D. This suggests that it might have a regulatory role in the browning program that is still unexplored. In addition, the *calponin 1 (CNN1)* gene which was reported in beige adipocytes was also found uniquely expressed in adipocytes C [44]. This gene was later reported to be abundantly present in isolated human brown preadipocytes [45].

Adipocytes D contained *FABP4*, also known as *adipocyte protein 2 (aP2)*, which is an adipogenic functional gene that is considered as an early marker of adipogenesis and is a downstream target of *PPAR-γ* [46]. This protein is an intracellular lipid chaperone that can reversibly bind to lipids to regulate lipid trafficking and transport to different organelles in the cell [47]. In addition, as an adipokine, *FABP4* regulates glucose and lipid metabolism when released into the bloodstream, acting as a humoral factor [48]. *FABP4* is known to be expressed in BATs [49]. It was shown that *FABP4* can increase thermogenesis in response to both a high-fat diet and cold exposure by promoting the intracellular conversion of thyroid hormones T4 to T3 in mice BATs. Also, elevated *FABP4* expression is observed in BAT of hibernating animals and cold-induced rodents [50]. The upregulation of this gene indicates more brown adipogenesis in D, though the gene was also expressed in C but with lower expression. *FABP4* is the target of *PPAR-γ* and for this reason, it is affected by thiazolidinediones [47]. *FABP4* delivers *PPAR-γ* agonists to the nucleus, thus affecting the transcription of genes that are involved in enhanced adipogenesis. It was observed previously that rosiglitazone induces increased transcription at the *FABP4* locus in mouse 3 T3-L1 adipocytes [51]. As mentioned above, *FABP4* is the only gene that is upregulated in D compared with B and C. This suggests that the expression of this gene is enhanced by the constant rosiglitazone treatment. This gene is so effective that it enriched several statistically significant GO terms; some of these GO terms are adipocytes and brown adipocytes related (Fig. 8). This validates our earlier finding that rosiglitazone enhances the brown adipogenesis [17].

GO and KEGG enrichment analyses confirmed rosiglitazone effect on the activation of PPAR, and regulation of PI3K-Akt signaling pathways in both treated groups C and D (Fig. 6B and Fig. 7B). The main enriched GO terms between C vs D and associate with LDs were upregulated in D (Supplementary File 4). LDs are lipid storage monolayer present in the adipocytes cytoplasm surrounded by scaffolding proteins that control lipid passage into and out of the droplets [52, 53]. *Perilipin 1 (PLIN1)* is an LD-associated protein and is highly abundant in brown adipose tissues [54, 55]. It promotes exercise-induced browning of muscle lipid, and its deficiency is observed in obese individuals [56, 57]. Noteworthy, the nuclear transcription factor *PPAR-γ* increases the activity of *PLIN1* by binding to its promoter [58]. Another upregulated gene in D is *protein tyrosine kinase 2 beta (PTK2B)* that regulates multiple signaling events as a member of the focal adhesion kinase (FAK) family. The increase in *PTK2B* protein expression was observed in cultured murine beige adipocyte differentiation. It appears that *PTK2B* has an essential rule in the thermogenic gene program in beige adipocytes through interacting with key adipogenic transcription factors such as *PPAR-γ* and *C/EBP-α* [59]. Evidently, *PTK2B* is involved in the activation of the MAP kinase signaling pathway by stimulating *JNK* and *ERK1/2* activity [60, 61]. *Fayyad* et al. found that MAPK pathway is upregulated and associated with rosiglitazone treatment which in turn enhanced the brown adipogenesis of iMSC3 [17]. Additionally, many ECM related GO terms were upregulated and enriched in adipocytes D (Fig. 7A). Cell-ECM interactions are reported to influence the formation of brown adipocytes and regulate their thermogenic capacity through over-expression of *UCP1* [62]. Furthermore, few other studies highlighted that BAT function is regulated by its ECM [24]. This might explain the enrichment of ECM related GO terms in adipocytes D compared to adipocytes C. Therefore, a better understanding of these interactions and their role in enhancing and regulating the thermogenic capacity is needed to further explore possible therapeutic targets. This data revealed that ECM is not only involved in the differentiation of iMSC3 into adipocytes but is also involved in the enhanced differentiation of iMSC3 into adipocytes due to rosiglitazone.

In addition to the DEGs, we identified *Zinc Finger and BTB Domain Containing 16 (ZBTB16)*, *Homeobox A4 (HOXA4)*, and *Krüppel-like Factor 15 (KLF15)* to be uniquely expressed in D (Supplementary File 3). *ZBTB16* is known as a novel regulator of adaptive thermogenesis and is associated with the increased mitochondrial biogenesis and expression of many brown adipogenic markers [63, 64]. *HOXA4* was reported as a potentially important positive regulator of brown adipogenesis during the adipogenic differentiation of immortalized murine pre-adipocyte cell line [65]. Whereas *KLF15* is a master regulator of adipocytes differentiation and fasting responses playing key roles in the regulation of glucose, lipids, and amino acids metabolism [66, 67]. However, its physiological role in BAT needs to be unraveled [66]. Hence, the unique expression of *ZBTB16* and *HOXA4* do indicate that rosiglitazone enhances brown adipogenesis. Moreover, many novel noncoding transcripts were detected in rosiglitazone treated adipocytes such as lincRNAs, miRNA, snRNA, or snoRNA. A recent transcriptome study on human WATs and BATs lncRNAs revealed the roles of these non-coding RNAs in brown adipogenesis [68]. Several lncRNAs found in this study appeared to be present in our data as well, specifically in adipocytes D. The specific roles of these transcripts during adipogenesis, particularly in human adipocytes, deserve further investigations. The non-coding RNAs might affect the epigenetic changes during the differentiation that might enhance the brown adipogenesis. Remarkably, rosiglitazone addition in both induction and maintenance media triggered the unique expression of *PR domain containing 16 (PRDM16)* in adipocytes D. This master transcriptional co-regulator has a crucial role in promoting the expression of brown adipocytes genes and repressing white selective genes, hence considered as one of brown adipocyte selective genes [69, 70]. *PRDM16* is also essential for the determination and function of beige adipocytes [69]. It was also presented that *PRDM16* modulates the switch between skeletal myoblasts and brown fat cells. This regulator binds to *PPAR-γ* and thus stimulates brown adipogenesis [70]. It also activates the expression of thermogenic genes by co-activating *PPAR-γ* and *PPAR-α* in adipocytes [71]. Interestingly, the *Trafficking Regulator of GLUT4 1 (TRARG1)* was also exclusively expressed in D. This is a positive regulator of insulin-stimulated *GLUT4* trafficking and insulin sensitivity that works in a PI3K/Akt-dependent manner [72], suggesting the regulation of the pathways in the presence of rosiglitazone.

The upregulation of *FABP4* and the unique expression of several coding and non-coding RNAs, induced by constant rosiglitazone treatment, enhanced the brown adipogenesis. These differentially and uniquely expressed genes and signaling pathways that are regulated in the presence of rosiglitazone treatment need further investigations to expand our knowledge about adipogenesis, specifically brown adipogenesis. The uniquely expressed genes and non-coding RNAs in D compared with B also suggest that rosiglitazone might regulate most of the genes at the post-translational modifications during brown adipogenesis.

## Conclusion

This study provided comparisons of the transcriptomic profile of iMSC3 and adipocytes differentiated with and without rosiglitazone treatment. Moreover, it offers a reliable collection of differentially and uniquely expressed genes associated with adipogenic processes. This transcriptomic study confirmed our previous findings about the roles of rosiglitazone in the regulation of PPAR and PI3K-Akt signaling pathways during brown adipogenesis. This huge collection of data promotes broader investigations of previously studied adipogenesis and obesity related genes. Further study should be focused on the proteomics analysis to find the differentially expressed proteins that can validate our transcriptomic findings and provide further insights into the roles of rosiglitazone in the brown adipogenesis. Moreover, further study using an animal model is needed to confirm the effects of rosiglitazone on in vivo brown adipogenesis.

## Methods

### Differentiation of iMSC3 into adipocytes

The iMSC3 cells differentiation into adipocytes with and without rosiglitazone was carried in our previous study [17]. Briefly, iMSC3 cells (abm T0529, Canada) were seeded into 6-well plates at a density of 5 × 10^4^ cells/well and maintained incomplete growth (MEM) media (Sigma-Aldrich M2279, USA) containing 10% Fetal Bovine Serum (FBS) (Sigma-Aldrich F6178, USA), 1% penicillin–streptomycin (Sigma-Aldrich P4333, USA) and 200 μM of L-glutamine (Sigma-Aldrich G7513, USA). The culture was incubated at 37 °C in a humidified incubator with 5% CO2. Prior differentiation, the cells at 70–80% confluency were subjected to serum starvation for 24 h. The differentiation was carried out in four experimental groups: (Group A) undifferentiated iMSC3, (Group B) iMSC3 differentiated into adipocytes without rosiglitazone treatment, (Group C) iMSC3 differentiated into adipocytes supplemented with 2 μM of rosiglitazone in induction media only, (Group D) iMSC3 differentiated into adipocytes supplemented with 2 μM rosiglitazone in induction and maintenance media throughout the differentiation period of 12 days. Adipogenic differentiation was induced by induction medium I (DMI) containing 0.5 μM/mL 3-isobutyl-1-methylxanthine (IBMX) (Sigma-Aldrich I17018, USA), 1 μM/mL Insulin (ITS) (Sigma-Aldrich I3146, USA), 0.25 μM/mL Dexamethasone (Sigma-Aldrich D4902, USA), 0.1 μM/mL Indomethacin (Sigma-Aldrich I7378, USA) with or without 2 μM/mL rosiglitazone (Sigma-Aldrich R2408, USA). After 48 h the media was changed to differentiation maintenance media II (DMII) containing 1 μg/mL insulin (ITS) with or without 2 μM/mL rosiglitazone for 2 days [73–77]. The two-step adipogenic differentiation protocol was repeated for 3 cycles for a total of 12 days. The differentiation experiment was repeated three times, ending with a total of three biological replicas and three technical replicas for each experimental condition. The iMSC3 and the differentiated adipocytes were harvested for further analysis.

### Nile red and DAPI staining of adipocytes

Nile red staining was performed following the protocol described by *Greenspan* et al. with modifications [78]. In brief, nile red stock solution of 1 mg/mL concentration was obtained by dissolving 5 mg of nile red powder (Sigma-Aldrich N3013, USA) in 5 mL of acetone. The stock solution was diluted to 1:100 nile red staining solution in 1 mM trizma-maleate (Sigma-Aldrich T3128, USA) and 3% w/v Polyvinylpyrrolidone (Sigma-Aldrich P2307, USA). The differentiated adipocytes were washed with PBS (Sigma-Aldrich D8537, USA) and fixed with 4% paraformaldehyde for 1 h. Then, the cells were stained with nile red to visualize the lipid droplets and DAPI (Life Technologies P36930, USA) to stain the nucleus. Olympus fluorescent microscope was used to observe the stained cells and imaged using cellSens Standard software.

### Oil-O red staining of adipocytes

Oil-O red staining was performed following *Aguena* et al. protocol with modifications [79]. The differentiated adipocytes were washed with PBS, fixed with 4% paraformaldehyde for 1 h, and incubated for 15 min at room temperature with 60% isopropanol solution. Then, the cells were air-dried, stained with Oil-O red staining solution (Sigma-Aldrich O1391, USA) (60% Oil-O Red stock solution and 40% distilled water) for 1 h, and washed with distilled water to remove excess stain. The stained cells were visualized under inverted microscope using Optika Vision Lite software.

### RNA extraction and quantification

Total RNAs were extracted from all experimental groups A, B, C, and D. The harvested cells were first lysed using Qiazol lysis reagent. Then, the extraction was carried out using miRNeasy extraction kit following the manufacturer’s instructions (Qiagen 217,004, Germany). The RNA quantity and purity were determined using Nanodrop 2000 spectrophotometer (Thermo Fisher Scientific, USA).

### RNA quality and integrity assessment

To guarantee the reliability of the data, quality control (QC) was performed at each step. RNA degradation and contamination were assessed on 1% agarose gel. Then, RNA purity was checked using NanoPhotometer spectrophotometer (Implen, USA). To obtain a quantitative assessment of RNA integrity and evaluate the RNA quantity, Agilent BioAnalyzer 2100 system was used. The samples concentration was first unified to 500 ng/ul, then proceeded with the Agilent RNA 6000 Nano Kit protocol as per the manufacturer’s instructions (Agilent Technologies 5067–1511, USA). The degradation was scored for each sample and represented as RNA Integrity Number (RIN) value. Only samples with RIN > 5.0 were used for subsequent library construction (Table S3).

### Library preparation for Transcriptome sequencing

A total amount of 1 μg RNA per sample was used as input material for the RNA sample preparations. NEBNext® UltraTM RNA Library Prep Kit for Illumina® (NEB, USA) was used to generate sequencing libraries following manufacturer’s recommendations. To attribute sequences to each sample, index codes were added. Briefly, poly-T oligo-attached magnetic beads were used to purify mRNA from total RNA. Fragmentation was carried out using divalent cations under elevated temperature in NEBNext First Strand Synthesis Reaction Buffer (5X). In order to select cDNA fragments, preferably of 150-200 bp in length, the library fragments were purified using AMPure XP system (Beckman Coulter, USA). Then, 3 μl of USER Enzyme (NEB, USA) was used with size-selected, adaptor ligated cDNA at 37 °C for 15 min followed by 5 min at 95 °C. After that, PCR was performed using Phusion High-Fidelity DNA polymerase, Universal PCR primers, and Index (X) Primer. Finally, the PCR products were purified (AMPure XP system) and library quality was assessed using the Agilent Bioanalyzer 2100 system.

### Clustering and sequencing

The clustering of the index-coded samples was performed on a cBot Cluster Generation System using PE Cluster Kit cBot-HS (Illumina, USA) according to the manufacturer’s instructions. After cluster generation, the library was sequenced using Illumina NovaSeq 6000 platform (Illumina, USA) and paired-end reads were generated in 300 cycles. All generated sequencing data are deposited in Gene Expression Omnibus (GEO) database with the accession number (GSE171826).

### Data analysis

#### Quality control

To ensure the quality and reliability of data analysis, the raw reads obtained in FASTQ format were first processed through fastp. Reads containing adapter sequences and poly-N sequences were removed from the raw data along with low-quality reads. Simultaneously, Q20, Q30 and GC content of the clean data were calculated. All the downstream analyses were performed based on high quality clean data.

#### Mapping to reference genome

Reference genome and gene model annotation files were directly downloaded from genome website browser (NCBI, UCSC, and Ensembl). Using STAR software, the paired-end clean reads were aligned to the reference genome. This software is based on a previously undescribed RNA-seq alignment algorithm that uses sequential maximum mappable seed search in uncompressed suffix arrays followed by seed clustering and stitching procedure. Compared to other RNA-seq aligners, STAR exhibits better alignment precision and sensitivity for both experimental and simulated data [80]

#### Quantification

The gene expression level was estimated by the abundance of transcript mapped to genome or exon. FeatureCounts was used to count the reads number mapped to each gene. The FPKM value was calculated for each gene based on the length and reads count mapped to this gene.

#### Differential gene expression analysis

Differential gene expression analysis between two groups A vs B, B vs C, C vs D, and B vs D, each having three biological replicates, was performed using DESeq2 R package. DESeq2 provides statistical routines for determining differential expression in digital gene expression data using a model based on the negative binomial distribution [81]. Using the Benjamini and Hochberg’s approach for controlling the False Discovery Rate (FDR), the resulting *P* values were adjusted. Genes with an adjusted *P* value < 0.05 found by DESeq2 were considered as differentially expressed for the three biological replica.

#### GO and KEGG enrichment analysis

Enrichment analysis enables us to attribute biological functions or pathways that are significantly associated with the DEGs. GO enrichment analysis of DEGs was implemented by the clusterProfiler R package. GO terms with corrected *P* value < 0.05 were considered significantly enriched by differential expressed genes. R package clusterProfiler was used to test the statistical enrichment of differential expression genes in KEGG pathways. Those terms with adjusted *P* value < 0.05 were considered as significantly enriched.

### cDNA synthesis and RNA-Seq validation by quantitative RT-PCR

cDNA was synthesized from the total RNA using QuantiTect Reverse Transcription kit following the manufacturer’s protocol (Qiagen 205,311, Germany). To confirm the transcriptome results, a total of 13 DEGs were selected and analyzed by qPCR using the same samples used for the RNA sequencing. The qPCR primer sequences for target genes are presented in (Table 2). QuantStudio3 Real-Time PCR System (Applied Biosystems, USA) was used to perform real-time gene expression study and using Maxima SYBR Green/ROX qPCR Master Mix (2x) (Thermo Fisher Scientific K0223, USA). *GAPDH* was used to normalize the obtained expression levels. The results were analyzed using Design and Analysis Software v1.5.1 (Thermo Fisher Scientific, USA), and the relative expression of genes was calculated using the 2^−ΔΔCT^ method [82].

**Table 2:** qPCR primer sequences of target genes used for RNA-Seq validation

**Gene Name**	**RT-PCR Primer Seq (Group A vs B)**		**References**
	**Forward primer (5′-3′)**	**Reverse primer (5′-3′)**	
Apolipoprotein E (APOE)	CTGCGTTGCTGGTCACATTCC	CGCTCTGCCACTCGGTCTG	[83]
WNT1 inducible signaling pathway protein 1 (WISP1)	GAAGCAGTCAGCCCTTATG	CTTGGGTGTAGTCCAGAAC	[84]
Rho GTPase activating protein 6 (ARHGAP6)	GGGAGGGAGGCATTCATCTAC	GTGGCCCACCAGCATAAAC	[85]
growth differentiation factor 15 (GDF15)	CCAAAGACTGCCACTGCATA	GAATCGGGTGTCTCAGGAAC	[86]
retinol binding protein 4 (RBP4)	TACTCCTTCGTGTTTTCCCGG	TAACCGTTGTGGACGATCAGC	[87]
**Gene Name**	**RT-PCR Primer Seq (Group B vs C)**		**References**
prostaglandin E receptor 2 (PTGER2)	AGGAGACGGACCACCTCATTC	GCCTAAGGATGGCAAAGACCC	[88]
snail family transcriptional repressor 1 (SNAI1)	GGTTCTTCTGCGCTACTGCT	TAGGGCTGCenunTGGAAGGTAAA	[89]
**Gene Name**	**RT-PCR Primer Seq (Group C vs D)**		**References**
insulin like growth factor binding protein 2 (IGFBP2)	GCCCTCTGGAGCACCTCTACT	CATCTTGCACTGTTTGAGGTTGTAC	[90]
ras related dexamethasone induced 1 (RASD1)	CCACCGCAAGTTCTACTCCAT	CCAGGATGAAAACGTCTCCTGT	[91]
fatty acid binding protein 4 (FABP4)	GCCAGGAATTTGACGAAGTCAC	TTCTGCACATGTACCAGGACAC	[92]
prostaglandin I2 synthase (PTGIS)	CTGGTTGGGGTATGCCTTGG	TCATCACTGGGGCTGTAATGT	[93]
apolipoprotein L3 (APOL3)	GCAAGGGACATGATGCCAGA	AAGAGTTTCCCCAAGTCAAGAGG	[94]
phosphatidylinositol-4-phosphate 3-kinase catalytic subunit type 2 beta (PIK3C2B)	CAGGCTTCAAGAGGCACTCA	TGGTCATCATTCACCGTCCG	[95]
GAPDH	AGGGCTGCTTTTAACTCTGGT	CCCCACTTGATTTTGGAGGGA	[17]

### Statistical analysis

All experiments were performed in triplicates and results were expressed as the mean ± standard deviation. Statistical significance was analyzed using GraphPad Prism 9 software to perform unpaired two-tailed student t-test considering significance at *P* value < 0.05.

## Supplementary Information

**Additional file 1.**

**Additional file 2.**

**Additional file 3.**

**Additional file 4.**

**Additional file 5.**

## Data Availability

All data generated and/or analyzed during this study are deposited in the GEO database NCBI (https://www.ncbi.nlm.nih.gov/geo/), with the accession number: GSE171826.
