# Supplementary material for: Transcriptomic profiling of the telomerase transformed Mesenchymal stromal cells derived adipocytes in response to rosiglitazone
Source: BMC Genom Data. 2022 Mar 9;23:17. doi: 10.1186/s12863-022-01027-z (PMC8905835; doi:10.1186/s12863-022-01027-z)
Supplement: Supplementary file 1 — Additional file 1. [file 12863_2022_1027_MOESM1_ESM.docx]

**Table-S1:** Data quality summary.

| Sample | raw_reads | clean_reads | raw_data(G) | clean_data(G) | error_rate(%) | Q20(%) | Q30(%) | GC_content(%) |
| --- | --- | --- | --- | --- | --- | --- | --- | --- |
| A1 | 20255196 | 20088844 | 6.1 | 6.0 | 0.02 | 98.06 | 94.34 | 46.49 |
| A2 | 20612719 | 20359951 | 6.2 | 6.1 | 0.02 | 98.26 | 94.86 | 50.06 |
| A3 | 23575247 | 23223228 | 7.1 | 7.0 | 0.03 | 97.96 | 94.20 | 50.00 |
| B1 | 22985639 | 22623772 | 6.9 | 6.8 | 0.02 | 98.09 | 94.43 | 49.74 |
| B2 | 30306017 | 29861490 | 9.1 | 9.0 | 0.03 | 97.86 | 93.92 | 50.10 |
| B3 | 30329819 | 29824479 | 9.1 | 8.9 | 0.03 | 97.50 | 93.09 | 49.81 |
| C1 | 29113672 | 28687246 | 8.7 | 8.6 | 0.02 | 98.02 | 94.35 | 49.89 |
| C2 | 22787408 | 22498856 | 6.8 | 6.7 | 0.02 | 98.07 | 94.49 | 50.00 |
| C3 | 29837960 | 29453296 | 9.0 | 8.8 | 0.02 | 98.02 | 94.37 | 50.02 |
| D1 | 24225292 | 23891032 | 7.3 | 7.2 | 0.02 | 98.09 | 94.48 | 49.79 |
| D2 | 27668752 | 27070097 | 8.3 | 8.1 | 0.03 | 97.70 | 93.47 | 48.75 |
| D3 | 23698668 | 23208202 | 7.1 | 7.0 | 0.03 | 97.75 | 93.69 | 50.24 |

(1) Sample name: sampleID.

(2) Raw reads: reads count from the raw data, four rows as a unit, with statistics of reads count for every sequencing.

(3) Clean reads: Clean data are reads count filtered from raw data. Statistics method is similar with raw reads. All the following analysis is based on clean data.

(4) Raw bases: Base number of raw data. (number of raw reads) * (sequence length), converting unit to G.

(5) Clean bases: Base number of raw data after filtering. (number of clean reads) * (sequence length), converting unit to G.

(6) Error rate (%): base error rate of whole sequencing.

(7) Q20(%): The percentage of the bases whose Q Phred values is greater than 20. (Number of bases with Q Phred value > 20) / (Number of total bases) *100.

(8) Q30(%): The percentage of the bases whose Q Phred values is greater than 30. (Number of bases with Q Phred value > 30) / (Number of total bases) *100.

(9) GC content (%): The percentage of G&C base numbers of total bases. (G&C base number) / (Total base number)*100.

**Table-S2:** Mapping results summary.

| Sample Name | A1 | A2 | A3 | B1 | B2 | B3 | C1 | C2 | C3 | D1 | D2 | D3 |
| --- | --- | --- | --- | --- | --- | --- | --- | --- | --- | --- | --- | --- |
| Total reads | 40177688 | 40719902 | 46446456 | 45247544 | 59722980 | 59648958 | 57374492 | 44997712 | 58906592 | 47782064 | 54140194 | 46416404 |
| Total mapped reads | 39236472 | 39801122 | 45174256 | 44118118 | 57998830 | 57674684 | 55863268 | 43838740 | 57346908 | 46589788 | 52295532 | 44857868 |
| Uniquely mapped reads | 38278058 | 38864706 | 44060598 | 43039912 | 56483464 | 56217466 | 54398810 | 42752816 | 55861626 | 45418738 | 51059514 | 43658978 |
| Multiple mapped reads | 958414 | 936416 | 1113658 | 1078206 | 1515366 | 1457218 | 1464458 | 1085924 | 1485282 | 1171050 | 1236018 | 1198890 |
| Total mapping rate | 97.66% | 97.74% | 97.26% | 97.5% | 97.12% | 96.69% | 97.36% | 97.42% | 97.35% | 97.5% | 96.59% | 96.64% |
| Uniquely mapping rate | 95.27% | 95.44% | 94.86% | 95.12% | 94.58% | 94.25% | 94.81% | 95.01% | 94.83% | 95.05% | 94.31% | 94.06% |
| Multiple mapping rate | 2.39% | 2.30% | 2.40% | 2.38% | 2.54% | 2.44% | 2.55% | 2.41% | 2.52% | 2.45% | 2.28% | 2.58% |

(1) Sample name: sampleID

(2) Total reads: total clean reads used for analysis

(3) Total mapped reads: numbers of reads being mapped on the genome, the ratio should higher than 70%.

(4) Uniquely mapped reads: numbers of reads being mapped on single position of the genome

(5) Multiple mapped reads: numbers of reads being mapped on more than one position of the genome.

(6) Total mapping rate: (mapped reads)/(total reads)*100

(7) Uniquely mapping rate: (uniquely mapped reads)/(total reads)*100

(8) Multiple mapping rate: (multiple mapped reads)/(total reads)*10

**Table-S3:** RIN values of the sequenced RNA samples.

| **Sample Name** | **Sample Details** | **RIN Value** |
| --- | --- | --- |
| **A1** | Undifferentiated iMSC3 | 6.7 |
| **A2** |  | 8.2 |
| **A3** |  | 8.6 |
| **B1** | iMSC3-derived adipocytes without rosiglitazone | 8.4 |
| **B2** |  | 9.5 |
| **B3** |  | 9.3 |
| **C1** | iMSC3-derived adipocytes under rosiglitazone treatment in induction media only | 8.5 |
| **C2** |  | 8.5 |
| **C3** |  | 6.9 |
| **D1** | iMSC3-derived adipocytes under rosiglitazone treatment both in the induction and maintenance media | 8.6 |
| **D2** |  | 8.5 |
| **D3** |  | 5.7 |
